# Draft Genome Sequence of *Corynebacterium amycolatum* Strain ICIS 53 Isolated from a Female Urogenital Tract

**DOI:** 10.1128/genomeA.01267-16

**Published:** 2016-11-10

**Authors:** Irina V. Gladysheva, Sergey V. Cherkasov, Yuriy A. Khlopko, Andrey O. Plotnikov, Natalya E. Gogoleva

**Affiliations:** a Institute for Cellular and Intracellular Symbiosis, Ural Branch of Russian Academy of Sciences, Orenburg, Russia; b Kazan Institute of Biochemistry and Biophysics of the Kazan Scientific Center of the Russian Academy of Sciences, Kazan, Russia

## Abstract

This report describes the draft genome sequence of *Corynebacterium amycolatum* strain ICIS 53, isolated from the reproductive tract of a healthy woman. The size of the genome was 2,460,257 bp (58.98% G+C content). Annotation revealed 2,173 coding sequences, including 2,076 proteins, 7 rRNA genes, and 53 tRNA genes.

## GENOME ANNOUNCEMENT

The genus *Corynebacterium* includes straight or slightly curved rod-shaped, Gram-positive, catalase-positive, nonspore-forming, nonmotile bacteria that belongs to the family *Corynebacteriaceae* of the phylum *Actinobacteria* (1). They are widely known pathogenic corynebacteria—causative agents of diphtheria and diphtheria-like diseases (2). In addition, there are nonpathogenic corynebacteria that constitute a significant part of the normal flora, especially on mucosa of the upper respiratory tract, urogenital tract, and in moist areas of human skin (3–6). The participation of particular strains of nonpathogenic corynebacteria in the protection of different biotopes of the human body from infections (7, 8) has been proven. Some strains of nonpathogenic corynebacteria have been recommended for use as probiotic microorganisms (9, 10). In addition, investigations are being conducted on the use of corynebacteria as immunomodulators in immunotherapy of tumors (11).

Here, we present a draft genome sequence of *Corynebacterium amycolatum* strain ICIS 53 isolated from the reproductive tract of a healthy woman. Our previous studies have showed that the strain has prospective probiotic properties such as antagonistic activity against pathogenic and opportunistic microorganisms, as well as the ability to enhance antagonistic activity of the peroxide-producing lactobacilli (12, 13).

Preparation of DNA libraries and sequencing was carried out at the Center of Shared Equipment “Persistence of Microorganisms” of the Institute for Cellular and Intracellular Symbiosis UrB RAS (Orenburg, Russia). Genomic DNA was extracted from an overnight culture of *C. amycolatum* ICIS 53 and used to prepare a DNA library with the Nextera XT DNA sample preparation kit (Illumina, San Diego, CA). The library was sequenced in a 2 × 300-nucleotide run using the MiSeq reagent kit version 3 and MiSeq desktop sequencer (Illumina). The reads were quality trimmed using the sliding window mode of the Trimmomatic program (14). *De novo* genome assembly was performed using the SPAdes genome assembler (St. Petersburg genome assembler, version 3.9.0) (15). The assembly yielded 45 contigs covering a total of 2,460,257 bp with an *N*_50_ of 170,644 bp, a G+C content of 58.98%, and an average coverage of 100. Four contigs were less than 200 bp and removed from the analysis. The genome sequence was annotated using the National Center for Biotechnology Information (NCBI) Prokaryotic Genome Annotation Pipeline (PGAP) (http://www.ncbi.nlm.nih.gov/genome/annotation_prok), which revealed 2,173 coding sequences, including 2,076 proteins, 34 pseudogenes, 7 rRNA genes (5S, 16S, and 23S), 53 tRNA genes, and 3 noncoding RNA (ncRNAs) genes.

Analysis of the strain *C. amycolatum* ICIS 53 genome revealed genes involved in resistance to antibiotics, such as vancomycin, fluoroquinolones, beta-lactam antibiotics, tolerance to oxidative stress, glycogen metabolism, and lactate fermentation. A number of genes are responsible for synthesis of possible biosurfactants, such as phospholipids, fatty acids, and neutral lipids. These properties may be crucial for the ability of this strain to survive in association with lactobacilli in the female reproductive tract and to prevent its colonization by pathogenic microorganisms.

### Accession number(s).

This whole-genome shotgun project has been deposited at DDBJ/ENA/GenBank under the accession number MIFV00000000. The version described in this paper is version MIFV01000000.
